# Matrix Stiffness Modulates 3D Spheroid-Derived Extracellular Vesicle Profiles and Discovery of Piezo1 Cargo

**DOI:** 10.1007/s12195-026-00931-z

**Published:** 2026-08-04

**Authors:** Maulee Sheth, Supasek Kongsomros, Manju Sharma, Maria Lehn, Takanori Takebe, Vinita Takiar, Trisha Wise-Draper, Somchai Chutipongtanate, Leyla Esfandiari

**Affiliations:** 1 Department of Biomedical Engineering, University of Cincinnati, Cincinnati, OH USA; 2 Division of Hematology/Oncology, Department of Internal Medicine, University of Cincinnati College of Medicine, Cincinnati, OH USA; 3 Division of Gastroenterology, Hepatology and Nutrition & Division of Developmental Biology, and Center for Stem Cell and Organoid Medicine (CuSTOM), Cincinnati Children’s Hospital Medical Center, Cincinnati, OH USA; 4 Department of Pediatrics, University of Cincinnati College of Medicine, Cincinnati, OH USA; 5 Department of Radiation Oncology, University of Cincinnati College of Medicine, Cincinnati, OH USA; 6 University of Cincinnati Cancer Center, Cincinnati, OH USA; 7 Department of Environmental and Public Health Sciences, University of Cincinnati, Cincinnati, OH USA; 8 Department of Electrical Engineering and Computer Science, University of Cincinnati, Cincinnati, OH USA

**Keywords:** Small extracellular vesicles, Cancer spheroids, Piezo1, Extracellular matrix, Mechanotransduction

## Abstract

**Background:**

Increased extracellular matrix stiffness is a defining mechanical feature of solid tumors, yet how it regulates extracellular vesicle-mediated intercellular communication remains poorly understood in three-dimensional tumor microenvironments. Here, we demonstrate that ECM stiffness mechanistically regulates extracellular vesicle (EV) cargo loading in oral squamous cell carcinoma spheroids.

**Methods and Results:**

Using a tunable three-dimensional spheroid culture platform, we show that increased matrix stiffness enriches tumorigenic and metastatic non-coding RNA transcripts in EVs.  At a functional level, stiffness-primed EVs influence recipient spheroid growth by modulating proliferation and apoptosis. Notably, our study reveals that EVs are enriched in parental biomolecular cargo, including the mechanosensitive Piezo1 ion channel and adhesion and stemness molecule CD44. Protein expression and small RNA sequencing analyses confirm the incorporation of these components into spheroid-derived EVs in a stiffness-independent manner.

**Conclusion:**

Together, our findings identify ECM stiffness as a mechanistic regulator of EV composition and establish EVs as biomechanical signaling vectors that further influence cell proliferation in three-dimensional microenvironments.

**Graphical Abstract:**

**Figure:**
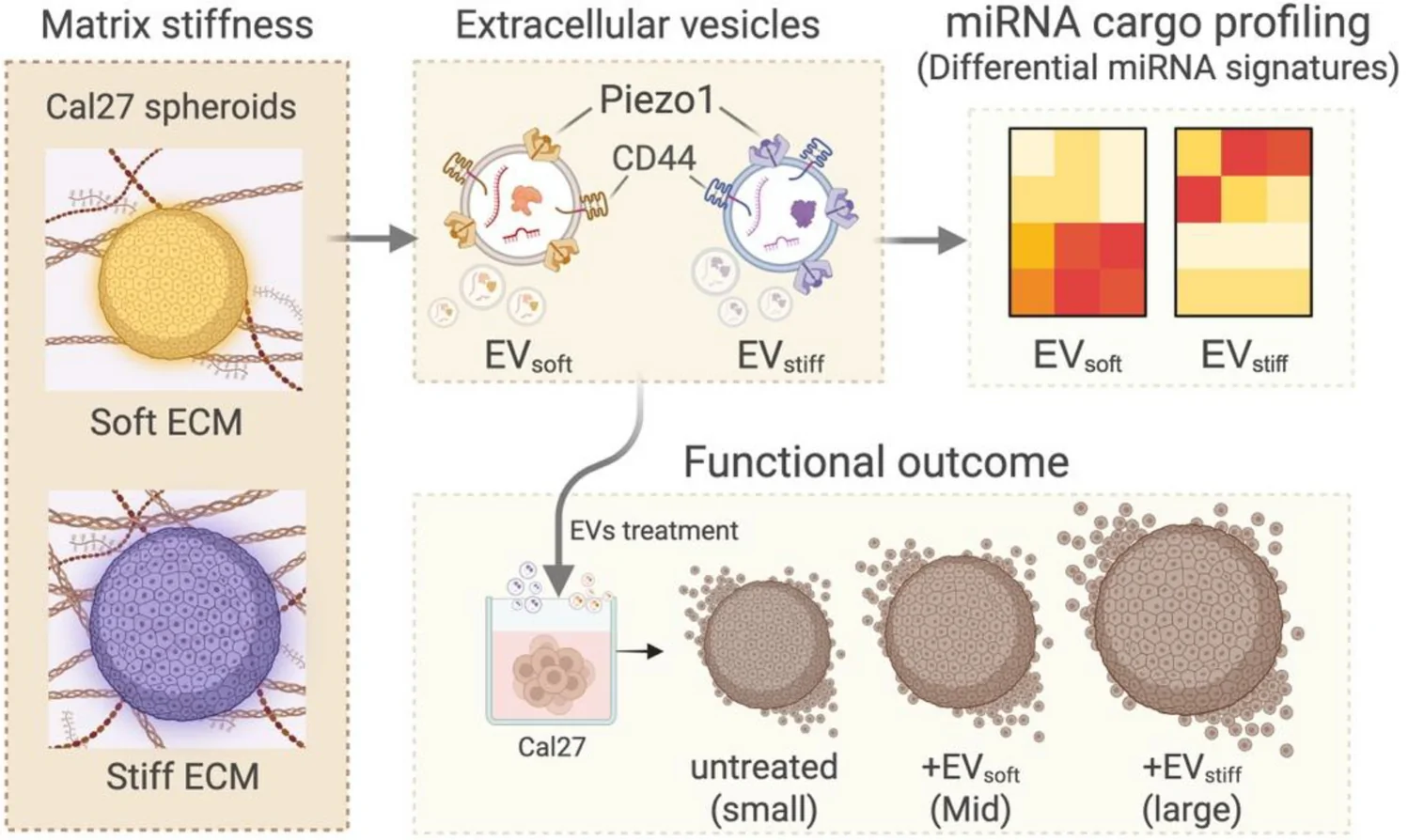

**Supplementary Information:**

The online version contains supplementary material available at https://doi.org/10.1007/s12195-026-00931-z.

## Introduction

Extracellular matrix, ECM, stiffening is a defining biophysical feature of many solid tumors and contributes to cancer progression by promoting proliferation, invasion, metastasis, and therapeutic resistance [1–3]. Cancer cells sense changes in ECM stiffness through mechanoreceptors, including integrins and mechanosensitive ion channels, which convert physical forces into biochemical signals that regulate cell behavior [4]. While the direct effects of ECM stiffness on tumor cells have been widely studied [1–3], less is known about how mechanical cues regulate intercellular communication, particularly through cell-secreted vesicles.

Extracellular vesicles (EVs) are membrane-bound vesicles released by most cell types that mediate intercellular signaling by transferring proteins, lipids, and nucleic acids to recipient cells. In cancer, EVs contribute to matrix remodeling, metastasis, and formation of the pre-metastatic niche [5–10]. Emerging studies suggest that ECM stiffness can also influence EV secretion and composition [11–13]. However, most of this work has been performed in two-dimensional cultures, which do not recapitulate the structural and mechanical features of native tissues. Simultaneously, it has been established that EVs derived from 2D cell cultures dramatically differ from those derived from 3D culture models, as monolayer systems fail to capture the crucial structural and topographical features of 3D in vivo microenvironments. In contrast, 3D culture models such as spheroids and organoids have been shown to improve EV yield, enhance therapeutic efficacy, and present more physiologically relevant molecular signatures [14, 15]. Despite these advantages, it remains unclear whether mechanical cues reshape EV molecular composition in ways that functionally influence tumor progression in relevant 3D contexts.

To investigate stiffness-regulated EV signaling in 3D environments, we employed Cal27 spheroids, a well-established model of oral squamous cell carcinoma (OSCC). OSCC, a major subtype of head and neck squamous cell carcinoma, is characterized by pronounced ECM remodeling and progressive tissue stiffening, both of which are strongly correlated with disease aggressiveness, metastasis, and poor clinical outcomes [16, 17]. A central mediator of cellular response to mechanical cues is the mechanosensitive ion channel Piezo1, which has emerged as a critical regulator of cancer cell proliferation, migration, and mechanotransduction across multiple tumor types [18]. Recent studies further suggest that ion channels are not restricted to the plasma membrane but can also be functionally associated with EVs, where they may influence vesicle biogenesis, cargo loading, and downstream signaling in recipient cells; for example, large conductance calcium-activated potassium channels were shown to influence the structural and functional properties of plasma-derived EVs [19]. These observations raise the intriguing possibility that mechanosensitive ion channels such as Piezo1 could be incorporated into EVs and contribute to the transmission of mechanically encoded signals within the tumor microenvironment.

Motivated by these gaps in knowledge, we investigated whether Piezo1 is present in EVs released from 3D OSCC spheroids and whether ECM stiffness modulates the cargo of spheroid-derived EVs. To the best of our knowledge, this is the first investigation of stiffness-mediated EV release in a 3D cancer culture system and the first to identify the presence of Piezo1 in EVs. Using a combination of protein expression analysis and transcriptomics, we demonstrate that ECM stiffness regulates the molecular composition of EV cargo, including non-coding RNAs, and that stiffness-primed EVs functionally modulate tumor cell proliferation in recipient spheroids. Collectively, these findings suggest that EVs act as carriers of mechanical information in 3D tumor microenvironments. This work not only provides new insight into how biomechanical cues are propagated between cancer cells but also highlights the potential of EV-associated mechanosensitive components as biomarkers or therapeutic targets for cancer progression.

## Results

### Spheroid-Derived EVs Carry Parental Piezo1 and CD44 Signatures Along with EV-Specific Markers

**Fig. 1:**
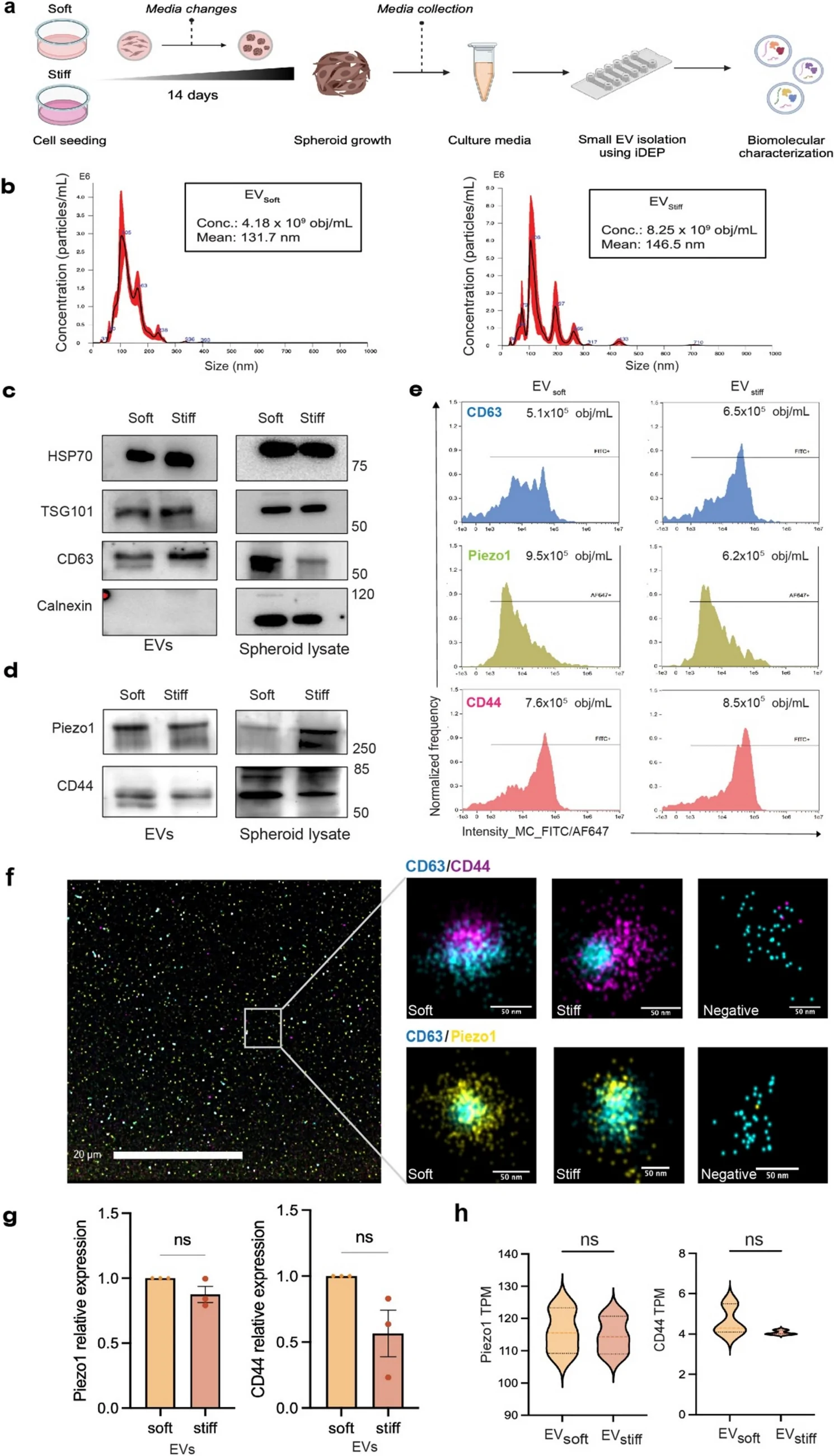
Spheroid-derived EVs contain CD44 and Piezo1 cargo along with EV-specific markers. **a** Workflow indicating the experimental timeline and procedure for spheroid culture and EV isolation. **b** Representative NTA graphs for purified EV_soft_ and EV_stiff_ samples. **c** Immunoblotting of EV markers (TSG101, HSP70, CD63) in spheroid lysates (SPL) and purified EVs from Cal27 spheroids cultured in soft and stiff matrices. Calnexin was used as a negative control. Molecular weights (in kDa) are shown to the right. **d** Immunoblotting of Piezo1 and CD44 in spheroid lysates (SPL) and purified EVs from Cal27 spheroids cultured in soft and stiff matrices. Unprocessed blots are available in the supplementary file. **e** Imaging flow cytometry for tetraspanin CD63 and new markers Piezo1 and CD44 reveal positive populations in purified EVs from both groups. The insert indicates positive objects/mL for each population. **f** Representative super-resolution microscopy field of view (left, scale bar = 20 microns) of captured and stained EVs. Single EVs analyzed as double-positive for CD63 and Piezo1/CD44 (right, scale bar = 50 nm). **g** Quantitative analysis of Piezo1 and CD44 protein levels was quantified using western blot between the two groups. n = 3 per group. Unpaired t-test. **h** Quantitative analysis of Piezo1 and CD44 mRNA presence quantified by transcripts per million (TPM) between the two groups. n = 3 per group. Unpaired t-test for Piezo1. Unpaired t-test with Welch’s correction for CD44

Cal27 human OSCC spheroids were cultured using the liquid overlay technique in Matrigel-based environments of defined stiffnesses, as described previously [20]. Hydrogels with elastic moduli of 0.3 kPa and 0.9 kPa, corresponding to soft and stiff conditions, represent the lower range of pathological stiffness observed in normal and cancerous oral tissues respectively (Supplementary Table S1) [16]. EVs were isolated from day 14 conditioned media using an insulator-based dielectrophoretic (iDEP) lab-on-a-chip platform that is more selective for the enrichment of small EVs below 200 nm (Fig. 1a) [21–25]. Nanoparticle tracking analysis showed an average concentration of 8.37 x 10^9^ particles/mL from spheroids cultured in stiff ECM, hereafter termed EV_stiff,_ and 4.34 x 10^9^ particles/mL from spheroids cultured in soft ECM, hereafter termed EV_soft_. (Fig. 1b). Mean EV diameters were similar between groups, measuring at 159.82 ± 5.20 nm for EV_soft_ and 160.43 ± 11.42 nm for EV_stiff_. Western blot analysis confirmed the presence of EV markers CD63, TSG101, and HSP70 and the absence of the endoplasmic reticulum marker calnexin (Fig. 1c), consistent with MISEV2023 guidelines [26].

We previously reported that matrix stiffness regulates the mechanosensitive ion channel Piezo1 and the stemness marker CD44 in Cal27 spheroids [20]. Here, we asked whether EVs derived from spheroids retain these parental protein markers. Western blot analysis confirmed the presence of Piezo1 and CD44 in EVs isolated from both soft and stiff conditions (Fig. 1d). We further validated the presence of these markers using imaging flow cytometry (iFCM) and direct stochastic optical reconstruction microscopy (dSTORM). iFCM, with a detection limit of approximately 70 nm, enabled bulk characterization of Piezo1 and CD44 positive EVs together with the tetraspanin marker CD63 (Fig. 1e). The gating strategy and controls are shown in Supplementary Fig. S1. Super-resolution imaging by dSTORM confirmed colocalization of Piezo1 or CD44 with CD63 in individual EVs (Fig. 1f). Visualization of Piezo1 and CD44 was further confirmed in EVs immobilized using biotinylated CD63 (Supplementary Fig. S2). Finally, the presence of Piezo1 and CD44 mRNA transcripts in EVs was confirmed by next-generation RNA sequencing (Fig. 1h).

Interestingly, both Piezo1 and CD44 protein and mRNA levels in spheroid-derived EVs were found to be independent of matrix stiffness (Fig. 1g, h). We confirmed this stiffness-independent expression of EV-Piezo1 by culturing Cal27 cells on 2D silicone-based substrates with Young’s moduli of 0.5 kPa and 8 kPa (Supplementary Fig. S3a, b). This 2D platform enabled us to remove the influence of confounding factors coming from a Matrigel-based model and to expand the stiffness range to more closely match that of squamous cell carcinoma tissue [16]. The cell-specific origin of Piezo1 in EVs was also validated using a knockout model, in which EVs secreted by genetically engineered Piezo1-KO Cal27 cells cultured on 0.5 kPa substrates lacked Piezo1 (Supplementary Fig. S3c). While Piezo1 levels in Cal27 cells increased from the 0.5 kPa to 8 kPa substrates, indicating stiffness-driven Piezo1 activation, EV-Piezo1 levels were found not to correlate with cellular Piezo1 and were independent of stiffness (Supplementary Fig. S3d, e). Taken together, our 2D and 3D data indicate that Piezo1 is present in these EV populations; however, unlike cellular Piezo1, its expression is not modulated by matrix stiffness.

## Matrix Stiffness Promotes Cancer-Associated Transcriptome in Spheroid-Derived EVs

**Fig. 2:**
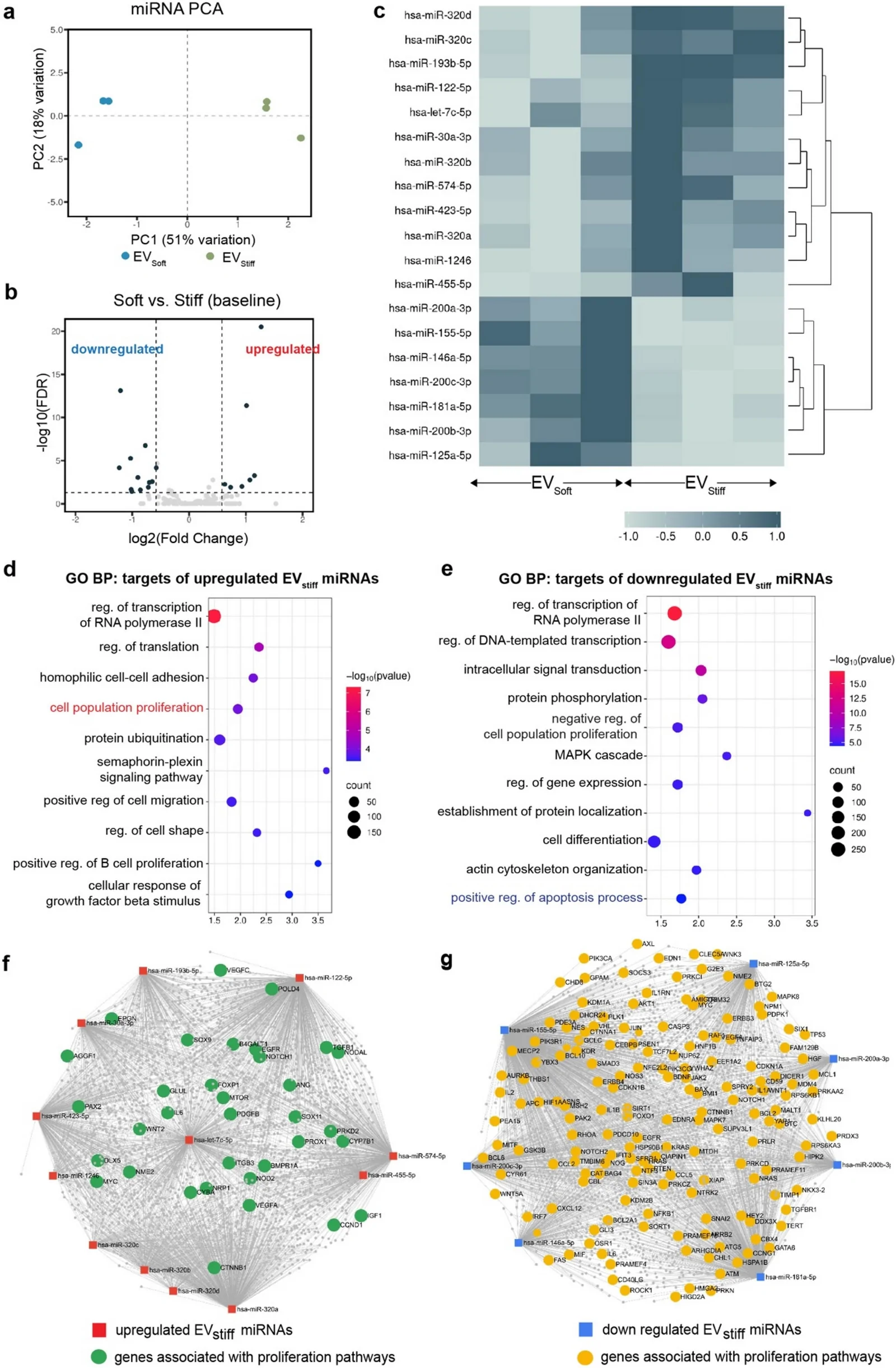
Effect of 3D matrix stiffness on miRNA composition of spheroid-derived EVs. **a** Principal component analysis of miRNA-seq wherein PC1 explains 51% of the variance and PC2 explains 18% of the variance. Blue circles indicate EV_soft_ and green circles indicate EV_stiff_ samples (n = 3 biological replicates each). **b** Volcano plot showing differentially expressed miRNAs (DEMs) (log2FoldChange > |0.58| and q-value <  = 0.05) between both groups with EV_stiff_ used as the baseline. Negative fold change indicates relatively increased expression in EV_stiff,_ and positive fold change indicates relatively decreased expression in EV_stiff_ quantified by miRNA sequencing. **c** Heatmap showing all 19 DEMs with hierarchical clustering. **d** Gene enrichment analysis of the target genes of miRNAs upregulated in EV_stiff_. **e** Gene enrichment analysis of the target genes of miRNAs downregulated in EV_stiff_. The size and color of the circles indicate the number of genes in the dataset associated with the corresponding pathway and the log10 P-value based on the color bar to the right, respectively. **f** miRNA-target network interaction analysis of miRNAs upregulated in EV_stiff_ and genes associated with proliferation pathways. **g** miRNA-target network interaction analysis of miRNAs downregulated in EV_stiff_ and genes associated with proliferation pathways. Squares indicate miRNAs and circles indicate genes associated with proliferation pathways

MicroRNAs (miRNAs) are important EV cargo that regulate intercellular and microenvironmental signaling in both physiological and pathological contexts. To determine whether matrix stiffness alters EV RNA cargo, we performed small RNA sequencing on EVs derived from spheroids cultured in soft and stiff matrices. Principal component analysis showed clear separation between EV_soft_ and EV_stiff_ samples, with PC1 and PC2 accounting for 51% and 18% of the variance, respectively (Fig. 2a). Differential expression analysis identified 19 miRNAs significantly modulated by stiffness, as shown in the volcano plot (Fig. 2b).

Among these differentially expressed miRNAs (DEMs) were frequently deregulated miRNAs with established diagnostic and prognostic relevance in HNSCC, including miR-320, miR-1246, miR-30a, and miR-574 [27–30] (Fig. 2c). Upregulated oncogenic DEMs in EV_stiff_ included miR-1246 which enhances tumor cell motility and invasion in OSCC, miR-30a-3p which regulates Akt signaling to promote metastasis, and miR-455-5p and miR-574-5p which are known as prognostic biomarkers [27–30]. Notably, miR-122-5p, which is detected uniquely in small EVs from HNSCC cell lines and promotes metastasis, was also upregulated in EV_stiff_ [31]. In contrast, tumor suppressor miRNAs included miR-200c-3p and miR-125a-5p known to be downregulated in metastatic patients and HNSCC cell line-derived small EVs, respectively, were decreased in stiff matrix conditions [32, 33]. Selected differentially expressed miRNAs were independently validated by quantitative real-time RT-PCR (Supplementary Fig. S4).

Gene ontology analysis of predicted targets of stiffness-regulated EV miRNAs revealed distinct functional enrichments. Targets of miRNAs upregulated in EV_stiff_ were significantly associated with biological processes including transcriptional regulation, translation, cell–cell adhesion, and cell population proliferation. Given the role of miRNAs in negatively regulating gene expression, enrichment of these miRNAs suggests potential suppression of pathways involved in cell growth (Fig. 2d). In contrast, targets of miRNAs downregulated in EV_stiff_ were enriched in intracellular signaling pathways, including the MAPK cascade, negative regulation of proliferation, and positive regulation of apoptosis. This suggests that the downregulation of these miRNAs may relive repression of target genes involved in apoptotic programs (Fig. 2e). Consistent with these findings, miRNA-target interaction network analysis revealed that these miRNAs organize into discrete and highly interconnected gene regulatory networks, with miRNA clusters converging on key hubs involved in proliferation pathways (Fig. 2f, g). Overall, these results indicate that ECM stiffness drives modulation of spheroid-derived EV miRNA cargo, targeting gene networks that regulate both growth-associated and apoptotic processes.

## Exogenous Addition of Stiffness-Primed EVs Influences Recipient Spheroid Size and Proliferation

**Fig. 3:**
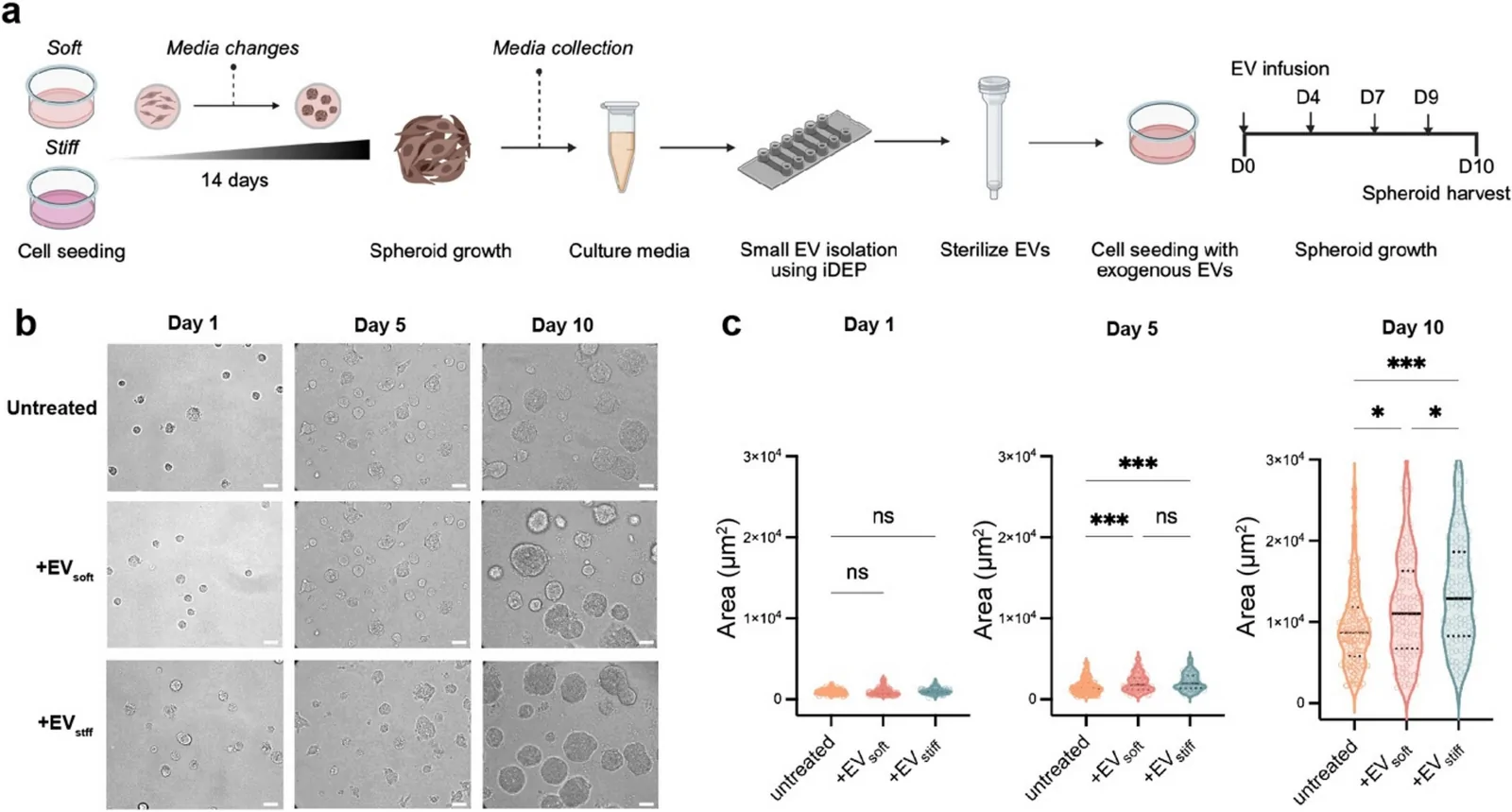
Exogenous addition of stiffness-primed EVs is associated with recipient spheroid enlargement. **a** Schematic of experimental workflow. EVs were isolated from spheroids cultured in soft or stiff ECM conditions for 14 days. The conditioned medium was collected, followed by EV isolation using the iDEP platform. EVs were sterilized and added exogenously (20 μg) to newly seeded spheroids at days 0, 4, 7, and 9 of culture. **b** Representative brightfield images of spheroid formation at days 1, 5, and 10 of untreated, + EV_soft_, and + EV_stiff_ conditions. Scale bars, 50 µm. **c** Quantification of spheroid area on days 1, 5, and 10. Data are presented as mean ± SEM. Statistical significance was determined by One-way ANOVA *p < 0.05, **p < 0.01; ns, not significant

Since differential miRNAs between EV_soft_ and EV_stiff_ were found to enrich pathways associated with proliferation, we sought to determine their functional downstream impact in modulating spheroid growth. Recipient spheroids in the same matrix conditions were treated with 20 ug EVs isolated from soft and stiff ECM spheroids, with PBS treatment serving as a control (Fig. 3a) [11]. At cell seeding on day 0, this EV dosing corresponded to ~ 10,000 EVs per cell across both treatment groups. Spheroid growth was monitored for ten days of culture in response to multi-point stiffness-primed EV treatment (Fig. 3b). Spheroids treated with EV_stiff_ indicated consistently increased area compared to control and EV_soft_-treated spheroids starting day 3. EV_soft_-treated spheroids, on the other hand, indicated increased area compared to control spheroids starting day 5, following two EV treatments (Supplementary Fig. S5). Overall, EV_stiff_-treated spheroids were the largest of all three treatment groups across the assay period. At the end of the culture duration on day 10, untreated spheroids had an average area of 9628.8 ± 486.5 μm^2^. This increased to 12067.6 ± 622.3 μm^2^ and 14415.8 ± 725.6 μm^2^ for EV_soft_ and EV_stiff_-treated spheroids, respectively (Fig. 3c).

**Fig. 4:**
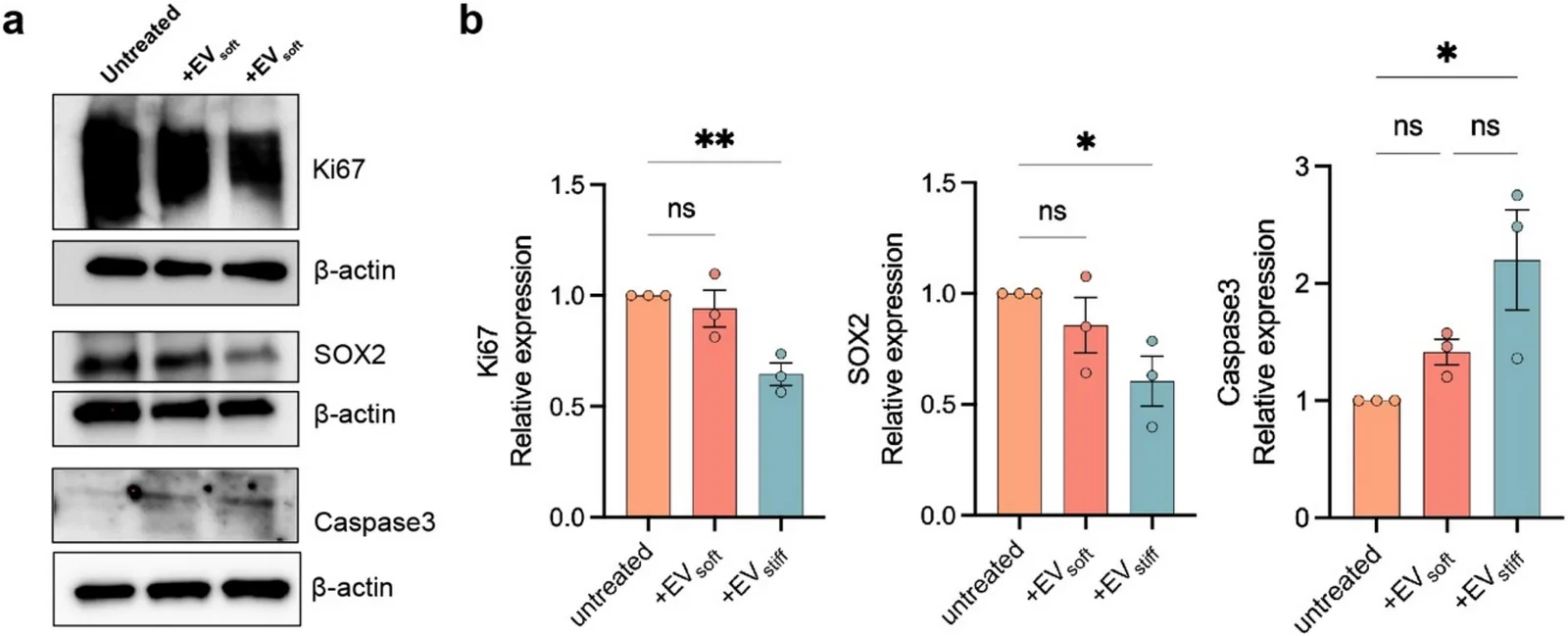
Exogenous addition of stiffness-primed EVs is associated with reduced proliferation in recipient spheroids. **a** Representative Western blot images showing Ki67, SOX2, and Caspase3 expression in each condition, with β-actin as a loading control. Unprocessed blots are available in the supplementary file. **b** Quantification of Ki67, SOX2, and Caspase3 band intensities normalized to β-actin and expressed relative to the untreated group. Statistical significance was determined by One-way ANOVA *p < 0.05, **p < 0.01; ns, not significant

Given that stiffness-primed EVs altered spheroid size and that differentially expressed miRNAs between the two groups were associated with proliferation and apoptosis-related pathways, we next assessed proliferative activity of the cells by evaluating Ki67 and SOX2 expressions as proliferation and stemness-associated markers respectively. Western blot analysis revealed no significant differences in Ki67 or SOX2 expression between untreated and EV_soft_-treated groups; however, EV_stiff_ treatment resulted in a significant reduction in both markers (Fig. 4a, b), indicating decreased cell proliferation despite increased spheroid size. We also quantified Caspase3, a protein associated with apoptosis [34], and found that it was significantly upregulated in EV_stiff_-treated spheroids (Fig. 4a, b), supporting increased cell death inside the core. These results support the miRNA-target gene ontology predictions and suggest that EVs from stiff ECM promote spheroid enlargement while decreasing the proportion of proliferative cells relative to apoptotic cells, consistent with expansion of non-viable regions within the spheroid core [35].

## Discussion

Extracellular matrix stiffness regulates the molecular composition of extracellular vesicles released from oral squamous cell carcinoma spheroids, suggesting that mechanical cues in the tumor microenvironment shape EV-mediated intercellular signaling in cancer. This study builds on our previous work showing that matrix stiffness remodels phenotypic and transcriptomic programs in Cal27 spheroids [20]. Three-dimensional spheroid models capture structural and mechanical features absent in monolayer cultures, including cell-cell interactions, spatial gradients, and matrix remodeling, all of which influence EV biogenesis and cargo selection. Furthermore, EVs derived from 3D cultures have been shown to exhibit molecular signatures more like *in vivo* circulating vesicles than EVs derived from 2D [15, 36]. Our findings extend this concept by demonstrating for the first time that the mechanical properties of the microenvironment further regulate the composition of 3D spheroid-derived EVs.

Our model used Matrigel at two different concentrations to generate mechanically distinct 3D environments that we have previously adopted to characterize stiffness-driven modulation of parental Cal27 spheroids. The utility of Matrigel in this platform comes with its set of trade-offs, as we and others have previously discussed [20, 37]. Importantly, since ECM stiffness is generally controlled by varying polymer concentrations, differences in gel microarchitecture represent confounding physical factors potentially influencing cell and EV behavior in addition to stiffness. Of relevance, the sub-micron effective pore size of Matrigel has been reported to allow for the selective diffusion of small EVs and soluble factors, while preventing the passage of larger particles such as apoptotic bodies, larger microvesicles, and oncosomes [38]. In line with this, isolation of spheroid-secreted EVs using the iDEP platform revealed an average particle size of < 180 nm for both groups, with purified EVs exhibiting field-defined EV-specific signatures.

A novel finding of our study is the identification of the mechanosensitive ion channel Piezo1 in spheroid-derived EVs. While we demonstrate that EV-associated Piezo1 originates from parental cells, validation across 2D and 3D conditions revealed that, unlike cellular Piezo1 expression, EV-associated levels remained unchanged across the stiffness range tested, indicating that vesicular Piezo1 abundance does not directly reflect stiffness-dependent changes in cellular expression. Such differences between cellular and EV protein levels are often attributed to selective cargo sorting mechanisms that vary with cell type and physiological context. While exact mechanisms governing this process are an emerging field of investigation, several factors have been proposed to influence such differential cargo profiles between cells and EVs [39–43]. For example, functional enrichment may drive preferential packaging of proteins and RNAs that enhance EV signaling roles. Cellular stress responses and extracellular environmental conditions can also shape EV composition by altering biogenesis and release under mechanical or biochemical stimuli. Simultaneously, membrane association dynamics also influence how transmembrane and membrane-bound proteins are incorporated into EVs. Finally, protein turnover and degradation processes may determine the retention and exclusion of certain cargo during EV formation. Together these observations suggest that the incorporation of Piezo1 into EVs is regulated independently of its cellular abundance and highlight the need to better understand the intracellular trafficking pathways governing EV membrane composition. Ion channels have only recently been implicated in EV biology, and their functional roles remain poorly defined; notably, Piezo1 is a mechanosensitive, non-selective cation channel overexpressed in several cancer types [44], with calcium signaling known to regulate vesicle trafficking, membrane fusion, and EV secretion [45]. While our study was not designed to directly assess whether Piezo1 mediates the functional effects of stiff matrix-derived EVs, its presence raises the possibility that mechanosensitive channels contribute to vesicular signaling, influencing intercellular communication. These findings point to a previously underexplored link between mechanotransduction and EV biology, suggesting that ion channel-associated signaling may represent an additional layer of regulation in mechanically driven tumor progression.

Given that matrix stiffness had elicited a profound influence on the transcriptomic composition of our spheroids, with ~ 5000 genes expressed differentially between the two groups as reported in our previous study [20], we further anticipated that RNA sequencing of the EV progeny secreted by these spheroids would reveal differential genetic profiles. We specifically focused on non-coding microRNAs due to their major roles in intercellular communication, ability to impact biological processes in recipient cells, and evident promise as diagnostic and prognostic tools in disease conditions [46, 47]. Of note, conflicting evidence exists regarding the impact of ECM stiffness on miRNA sorting into EVs, with one study reporting stiffness-mediated regulation of cellular miRNA expression and their sorting into EVs, while another stating that ECM stiffness primarily affects the sorting of lipids and proteins into EVs, with relatively minor influence on miRNA content [48–50]. Transcriptomic profiling revealed that stiffness indeed reshapes spheroid EV miRNA cargo in our samples. EVs derived from stiff matrices were enriched in miRNAs associated with tumor progression, while several tumor suppressive transcripts were reduced, consistent with established roles of these EV transcripts as oncogenes and tumor suppressors in head and neck cancers [46, 47].

Pathway analysis of target genes associated with differentially expressed EV miRNAs identified gene networks involved in regulating cell proliferation and apoptosis, with miRNA-target gene network analysis identifying highly interconnected proliferation-related hubs. This offered the premise that stiffness-primed EVs may function to regulate recipient cell behavior by negatively modulating proliferation and apoptotic programs. Since the target genes of miRNAs upregulated in EV_stiff_ were enriched in cell population proliferation pathways, we anticipated reduced proliferation in spheroids treated with EV_stiff_. At the same time, enrichment of apoptosis-related pathways among the target genes of miRNAs downregulated in EV_stiff_ suggested increased cell death in spheroids treated with EV_stiff_. We tested this functionality of stiffness-primed EVs in our 3D spheroid system. While EVs derived from stiff matrix spheroids promoted an increased average size of recipient spheroids, these spheroids were also characterized by reduced proliferation and increased apoptosis, corroborating our transcriptomic predictions with phenotypic outcomes. Increased spheroid size generally imposes diffusion limitations in oxygen and nutrient availability, resulting in spatial organization with a proliferative outer and necrotic inner zone. While our results represent marker abundance at a population level, and not spatially, larger spheroids are known to exhibit expanded necrotic regions and increased quiescent cell populations [51–53]. Taken together, our results indicate that stiffness-conditioned spheroid EVs carry bioactive cargo driving spheroid enlargement, which is further marked by reduced cell proliferation and the presence of a necrotic core population. These findings support a model in which EVs participate in propagating stiffness-associated transcriptional states within the tumor microenvironment, reinforcing cell–EV crosstalk as a component of mechanically driven tumor progression.

## Conclusion

This study identifies ECM stiffness as a regulator of EV cargo composition in a 3D oral squamous cell carcinoma spheroid model. We detect the mechanosensitive ion channel Piezo1 and the adhesion-related marker CD44 in spheroid-derived EVs and confirm the cellular origin of EV-associated Piezo1 using knockout controls. Stiff matrix EVs exhibited altered noncoding RNA profiles linked to cell proliferation pathways. Consistent with this, EVs produced under stiff matrix conditions enhanced the growth of recipient spheroids by modulating proliferation and apoptosis, demonstrating that stiffness-driven EV cargo differences have functional consequences in this model. Overall, our study supports the emerging view that the mechanical properties of the tumor microenvironment actively shape EV-mediated communication and reinforce the importance of studying EV biology in physiologically relevant three-dimensional systems. This work opens several important directions for future research. Defining the mechanisms that link stiffness sensing to EV biogenesis and cargo selection, including the basis for incorporation of mechanosensitive membrane proteins such as Piezo1, will be essential for understanding how mechanical cues are propagated within the tumor microenvironment. Importantly, our findings position EV-associated mechanosensitive components as potential biomarkers of tumor mechanical state and disease aggressiveness, and point to new opportunities to therapeutically target mechanically driven cancer signaling.

## Materials and Methods

### Cell Culture and Maintenance

Cal27 cells (ATCC, Manassas, VA) were cultured in DMEM media (Corning, San Diego, CA) containing 10% fetal bovine serum (Fisher Scientific, Waltham, MA) that was super-depleted of extracellular vesicles via 18-hour ultracentrifugation at 100,000xg [31], 1% penicillin-streptomycin (Fisher Scientific, Waltham, MA), 1% L-glutamine (Fisher Scientific, Waltham, MA), 1% sodium pyruvate (Fisher Scientific, Waltham, MA), and 1% non-essential amino acids (Fisher Scientific, Waltham, MA).

## 3D Cell Culture

Cal27 spheroids were generated using the media overlay technique [20]. This method plates cells in media containing a small amount of Matrigel on top of solidified gels to enable cell penetration into the gel for spheroid formation. 24-well plates were coated with a thin layer of 3 or 12 mg/mL Growth factor reduced Matrigel (Corning, San Diego, CA) prepared by dilution in ice-cold PBS and incubated for 30 min to allow the gel to solidify. Batch-to-batch variability of Matrigel was accounted for by utilizing the same batch for replicate experiments. Cal27 cells were trypsinized and counted for seeding. 10,000 cells in 500 μL media containing 1% Matrigel were plated on top of well coatings. Spheroids were cultured at 37 °C and 5% CO_2_ with regular media changes after the first 96 h and every 48 h after that. Day 14 spheroid culture media was stored at − 80 °C for purification of EVs.

## 2D Cell Culture

For 2D experiments, CytoSoft 6-well plates with elastic modulus 0.5 and 8 kPa (Advanced BioMatrix, Carlsbad, CA, USA) were used. Plates were coated with 100 ug/mL PureCol Type I collagen (Advanced BioMatrix, Carlsbad, CA, USA) for 1 hour at room temperature to functionalize the hydrophobic surface. Post-incubation, the remaining material was aspirated and coated surfaces were rinsed with PBS. Cal27 wildtype and Piezo1-KO cells in EV-depleted media were trypsinized, and 50,000 cells in 2.5 mL media were plated per well for five days of culture.

## CRISPR Piezo1 Knockout Cell Cloning

To generate Piezo1 knockout (KO) cell lines, single guide RNAs (sgRNAs) ACGCTTCAATGCTCTCTCGC targeting the second exon of mouse Piezo1 as reference were used. Cal27 cells were electroporated using Cas9-SgRNA (Sg697 and Sg698) complex to create a Piezo1 KO cell line by removing the 5^th^ exon and part of the adjacent intron using the Neon electroporator. Single-cell clones were genotyped using primers VS9720/9721. Cells were plated at single-cell density after a portion underwent bulk analysis to check for editing, then genotyped for the presence of KO alleles and the lack of a WT allele. Successful knockout clones were verified using Sanger sequencing and western blotting (Supplementary Dataset S3).

## Isolation of Extracellular Vesicles

EVs were isolated from culture media samples using an insulator-based dielectrophoresis approach [21, 22]. Culture media samples were centrifuged at 21,000xG at 4 °C for 20 minutes, and the supernatant was utilized for EV extraction. For purification from 2D cell culture media, conditioned medium was concentrated ~ 10-fold before extraction using Amicon Ultra-4 centrifugal filters (Millipore, Burlington, MA, USA). Briefly, glass micropipettes were backfilled with 1X filtered PBS buffer using a 33-gauge Hamilton syringe needle and positioned on a substrate. 50 μL sample and 1X filtered PBS were loaded at the tip side and base side chamber of the micropipette, respectively. EVs were trapped at the tip by applying a 10 V/cm direct current (DC) for 15 min, followed by a release in 15 μL 1X filtered PBS by reversing the applied voltage for another 10 min. 2 mL culture media samples were simultaneously processed by performing parallel aliquots to purify EVs in 600 μL PBS. Purified samples were stored at − 80 °C for further analysis. All EV replicate samples throughout the manuscript represent independent isolations from the conditioned media collected from three or more separate cell culture experimental repeats.

## Nanoparticle Tracking Analysis

Purified EVs were diluted in filtered PBS at a dilution of 1:20 and analyzed by nanoparticle tracking analysis (NTA) using a NanoSight NS300 (Malvern, Worcestershire, UK) and the NTA 3.1 software. Camera level 14 and detection threshold 5 were used for instrument settings. Five 60-second recordings were obtained per sample. All post-acquisition functions were at default settings to output the mean, mode, standard deviation, and estimated concentration for each particle size.

## Western Blot

Spheroid, cell, or EV samples (5 μg protein) were lysed with 1X RIPA buffer and mixed with 4X Leammli buffer (Bio-Rad, Hercules, CA, USA). 30 to 50 μg total protein was loaded for EV-Piezo1 detection, and 10 μg total protein was loaded for downstream targets following functional assays. The samples were heated at 95 °C for 5 min and then run into the NuPAGE™ 12%, Bis-Tris, 1.0 mm, Mini-PROTEAN TGX precast gels for 50 min (Bio-Rad, Hercules, CA, USA) before being transferred onto a PVDF membrane using a turboblot (Bio-Rad, Hercules, CA, USA). The membrane was blocked with Everyblot blocking buffer (Bio-Rad, Hercules, CA, USA) and then probed with 1:1000 anti-CD63 (Abcam, Waltham, MA, USA), anti-HSP70 (Abcam, Waltham, MA, USA), anti-TSG101 (Abcam, Waltham, MA, USA), anti-Piezo1 (Novus Biologicals, Centennial, CO, USA), anti-CD44 (Abcam, Waltham, MA, USA), anti-Calnexin (Abcam, Waltham, MA, USA), anti-Ki67 (Abcam, Waltham, MA), anti-SOX2 (Abcam, Waltham, MA, USA) or anti- β-actin (Abcam, Waltham, MA) at 4 °C overnight. After washing, the membranes were incubated with 1:2000 goat anti-rabbit IgG secondary antibody HRP (Abcam, Waltham, MA, USA) or and goat anti-mouse (Abcam, Waltham, MA) secondary antibodies as appropriate, at room temperature for 1 hour. The immunoblot was developed using Clarity Western ECL Substrate (Bio-Rad, Hercules, CA, USA) and imaged on a ChemiDoc MP Imaging system (Bio-Rad, Hercules, CA, USA). Densitometry for western blotting was quantified using Image J (Fiji; National Institutes of Health, Bethesda, MD, USA). Bands for 8-bit images were selected by a rectangular selection and plotted. Areas were measured by the wand tool and normalized for statistical analyses. For relative expression, protein bands were normalized to β-actin and quantified relative to the untreated control.

## Super-Resolution Microscopy

Sub-diffraction limit resolution images of single EVs were obtained using a Nanoimager S Mark II microscope (Oxford Nanoimaging, Oxford, UK) equipped with a 100X, 1.4 NA oil immersion objective, an XYZ closed-loop piezo 736 stage, and dual or triple emission channels split at 640 and 555 nm. ~ 1 x 10^9^ EVs in 1 mL were stained with 5 μg/mL mix of antibodies including anti-CD63-Cy38 (Oxford Nanoimaging, Oxford, UK) and anti-Piezo1-FITC (Biolegend, San Diego, CA, USA) conjugated using FITC conjugation kit (Abcam, Waltham, MA, USA) or anti-CD44-AF647 (Biolegend, San Diego, CA, USA). Antibody mix-only samples were acquired as negative controls. CD63 + Piezo1 samples were processed according to the manufacturer’s instructions to immobilize the stained EVs on chips provided with the EV profiler kit. CD63 + CD44 samples were prepared using a modified protocol. 5 μg/μL of antibody mix was mixed with 1 x 10^9^ EVs in PBS 0.2% bovine serum albumin in a final volume of 100 μL, overnight on a shaker at 4 °C. Following completion, the samples were purified using an Ultracel-100 K membrane filter (Merck Millipore, Burlington, MA, USA) and washed 3X with filtered PBS at 5000 RPM for 3 minutes. For imaging, μ-Slide 8-well glass-bottom slides (IbiDi, Fitchburg, WI, USA) were washed 2X with distilled water, then with 1 M potassium hydroxide, and finally with distilled water to clean the surface. For surface coating, 100 μL poly-L-lysine solution (Sigma Aldrich, St. Louis, MO, USA) was added and incubated at 37 °C for 2 hours. Upon incubation, the solution was removed carefully, and the sample of antibodies-EVs was added at a final volume of 100 μL and kept at 4 °C overnight. Next day, the buffer was removed, washed, and fixed with 3.7% formaldehyde for 15 minutes at 4 °C. Samples were finally washed 3X with filtered PBS. Ten to fifteen fields of view were recorded for each sample using direct stochastical optical reconstruction microscopy (dSTORM). Analysis was performed using algorithms including filtering, drift correction, and DBScan clustering developed by ONI (Oxford Nanoimaging, Oxford, UK) via the Collaborative Discovery (CODI) platform.

## Imaging Flow Cytometry

Advanced imaging flow cytometry of purified EVs was performed using an ImageStream^X^ Mark II imaging flow cytometer (Amnis, Seattle, WA, USA) at the CCHMC Research Flow Cytometry Core. Fifteen microlitre EV isolates were diluted in 15 μL 1X filtered PBS for flow acquisition. Samples were single stained with 1:20 CD63-FITC (Biolegend, San Diego, CA, USA), CD44-FITC (Biolegend, San Diego, CA, USA), Piezo1-AF647 (Novus Biologicals, Centennial, CO, USA), Isotype IgG1-FITC (Biolegend, San Diego, CA, USA), or Isotype IgG2b-AF647 (Biolegend, San Diego, CA, USA) in the dark for 30 minutes. Unstained, isotype, and antibody-only samples were acquired as negative controls. Data acquisition was performed with low-speed fluidics, high sensitivity, and 7-μm core size at 60X magnification. Channels Ch01 and Ch09 were used for brightfield, and Ch12 was used for side scatter. Data were collected for 3 min/sample for all samples. Analysis was performed using IDEAS software (Amnis, Seattle, WA, USA).

## Exogenous Addition of Stiffness-Primed EVs

For functional testing, purified EVs from both groups were sterilized using 0.22 μm centrifugal filters and measured for their total protein concentration. Recipient spheroids were cultured as described previously using 6 mg/mL Growth factor-reduced Matrigel (Corning, San Diego, CA). 20 μg EVs were exogenously added to spheroids four times (on days 0, 4, 7, and 9) over a period of ten days [11]. PBS-treated groups were used as baseline controls. Spheroid growth was assessed by brightfield microscopy for at least n = 100 spheroids per group and analyzed using ImageJ (Fiji; National Institutes of Health, Bethesda, MD, USA).

## RNA Isolation

Total RNA was extracted from purified EVs or bulk spheroids using the miRNeasy Micro kit (Qiagen, Valencia, CA) as per the manufacturer’s suggested protocol. Size distribution and estimated RNA concentration were measured using the Agilent 6000 Pico Kit using Bioanalyzer.

## Whole RNA Sequencing

Directional whole exosome RNA-seq was performed by the Genomics, Epigenomics and Sequencing Core (GESC) at the University of Cincinnati using established protocols as described previously with updates [54, 55]. To summarize, the quality of total RNA was quality control analyzed by Bioanalyzer (Agilent, Santa Clara, CA). A total of ~ 10 ng RNA was input for library preparation using NEBNext Ultra II Directional RNA Library Prep kit (New England BioLabs) under PCR cycle number 9. Library quality control and quantification were performed via Qubit quantification (ThermoFisher, Waltham, MA), and individually indexed libraries were proportionally pooled and sequenced using NextSeq 2000 Sequencer (Illumina, San Diego, CA) under the PE 2x61 bp setting to generate about 44 M reads. Upon sequencing completion, fastq files were generated via Illumina BaseSpace Sequence Hub for downstream data analysis.

## mRNA-Sequencing Analysis

RNA-seq reads in FASTQ format were first subjected to quality control to assess the need for trimming of adapter sequences or bad quality segments. The programs used in these steps were FastQC v0.11.7 [56], Trim Galore! v0.4.2 [57] and cutadapt v1.9.1 [58]. The trimmed reads were aligned to the reference human genome version hg38 with the program STAR v2.6.1e [59, 60]. Aligned reads were stripped of duplicate reads with the program sambamba v0.6.8 [60]. Gene-level expression was assessed by counting features for each gene, as defined in the NCBI's RefSeq database [61]. Read counting was done with the program featureCounts v1.6.2 from the Rsubread package [62]. Raw counts were normalized as transcripts per million (TPM). Differential gene expressions between groups of samples were assessed with the R package DESeq2 v1.26.0 [63]. Gene list and log2 fold change are used for GSEA analysis [64] using GO pathway dataset.

## miRNA Sequencing

MicroRNA-seq was performed by the GESC at the University of Cincinnati [65, 66]. For library preparation, NEBNext Small RNA Sample Library Preparation kit (NEB, Ipswich, MA) was used with a modified approach for precise miRNA library size selection, which makes it possible for the kit to process low input (ng level) and low-quality (Bioanalyzer RIN value < 3) RNA with better library recovery and miRNA reads alignment. Specifically, using ~ 8 ng total RNA as input, after 15 cycles of final PCR, libraries with unique indices were first equal-10 µl pooled, column cleaned up, and mixed with a custom-designed DNA ladder that contains 135 and 146 bp purified PCR amplicons. This size range corresponds to a miRNA library with 16-27 nt insert that covers all miRNAs. After high-resolution agarose gel electrophoresis and gel excision, the library pool ranging from 135 to 146 bp, including the DNA marker, was purified and quantified by NEBNext Library Quant kit (NEB) using QuantStudio 5 Real-Time PCR System (Thermofisher, Waltham, MA). The first round of sequencing was performed on NextSeq 2000 sequencer (Illumina, San Diego, CA) to generate a few million reads to quantify the relative concentration of each library. The volume of each library was then adjusted to generate the expected number of equal reads [~4.5 M read clusters] from each sample for final data analysis.

## miRNA-Sequencing Analysis

The exceRpt pipeline [62] was utilized for quality control of small RNA samples. Gene-level expression was assessed by counting features for each gene, as defined in the NCBI's RefSeq database [63]. Raw counts were normalized as transcripts per million (TPM).

For RNA target analyses, the two primary clusters representing upregulated and downregulated miRNAs in EV_stiff_ versus EV_soft_ were selected for downstream analysis. Target gene prediction was performed using miRDB [67], and only targets with a prediction score ≥ 80 were retained for further analysis [68]. Predicted target genes were submitted to DAVID (http://david.ncifcrf.gov) for gene ontology (GO) analysis to identify the enriched biological process categories ranked by enrichment score, with statistical significance set at P ≤ 0.05. Data visualization was performed using SRplot [69], and miRNA–target interaction networks were constructed using miRNet 2.0 [70].

## qRT-PCR

miRNA expression was quantified using the miRCURY LNA SYBR Green system (QIAGEN, Germantown, MD). Total RNA (0.5 ng per 10 µl RT reaction) was reverse transcribed into cDNA with the miRCURY LNA RT Kit (QIAGEN, Germantwon, MD), including the UniSp6 RNA spike-in as an exogenous control. The cDNA was diluted 1:30 and amplified using the miRCURY LNA SYBR Green PCR Kit (QIAGEN, Germantown, MD) with target-specific primers for hsa-miR-1246, hsa-miR-122-5p, hsa-miR-125a-5p, and hsa-miR-146a-5p (Primer assay, QIAGEN, Germantown, MD). Each 10 µl PCR reaction contained 5 µl of 2 × SYBR Green Master Mix, 1 µl of primer assay, 1 µl of nuclease-free water, and 3 µl of diluted cDNA. Amplification was performed in StepOne Real-Time PCR System (Applied Biosystems, Carlsbad, CA) using the following cycling conditions: 95 °C for 2 min, followed by 60 cycles of 95 °C for 10 s and 56 °C for 60 s, with a melt-curve analysis. Non-template controls (NTCs) were included as a negative control. Relative expression levels were determined using the ΔΔCt method, normalized to U6 snRNA, with EV_soft_ as the comparator group.

## Statistics

Data points are indicated as individual biological replicates and represented as mean ± SEM in the graphs. All statistical analyses between the two groups were conducted using an unpaired two-tailed t-test. Functional testing analyses between three groups were performed using one-way ANOVA. No outliers were removed from the analysis. All significant and non-significant p-values are indicated on the graphs with a p-value of ≤ 0.05 considered significant. Statistical analyses were performed using GraphPad Prism Ver 10.3.1 (GraphPad Software, San Diego, CA, USA).

## Supplementary Information

Below is the link to the electronic supplementary material.

Supplementary material 1 (DOCX 4201 kb)

## Data Availability

RNA-sequencing data have been deposited with the Accession No. GSE288252. The data supporting this study's findings are available within the article and its supplementary material.
