# Supplementary material for: Matrix Stiffness Modulates 3D Spheroid-Derived Extracellular Vesicle Profiles and Discovery of Piezo1 Cargo
Source: Cell Mol Bioeng. 2026 Aug 4;19(4):519–33. doi: 10.1007/s12195-026-00931-z (PMC13562468; doi:10.1007/s12195-026-00931-z)
Supplement: Supplementary file 1 [file 12195_2026_931_MOESM1_ESM.docx]

**Supporting Information for**

**Matrix stiffness modulates 3D spheroid-derived extracellular vesicle profiles and discovery of Piezo1 cargo**

Maulee Sheth^a#^, Supasek Kongsomros^a#^, Manju Sharma^a#^, Maria Lehn^b^, Takanori Takebe^c,d^, Vinita Takiar^e,f^, Trisha Wise-Draper^b,f^, Somchai Chutipongtanate^g,^, Leyla Esfandiari^a,f,g,i*^

^a)^ Department of Biomedical Engineering, University of Cincinnati, Cincinnati, OH, USA

^b)^ Division of Hematology/Oncology, Department of Internal Medicine, University of Cincinnati College of Medicine, Cincinnati, OH, USA

^c)^ Division of Gastroenterology, Hepatology and Nutrition & Division of Developmental Biology, and Center for Stem Cell and Organoid Medicine (CuSTOM), Cincinnati Children’s Hospital Medical Center, Cincinnati, OH, USA

^d)^ Department of Pediatrics, University of Cincinnati College of Medicine, Cincinnati, OH, USA

^e)^ Department of Radiation Oncology, University of Cincinnati College of Medicine, Cincinnati, OH, USA

^f)^ University of Cincinnati Cancer Center, Cincinnati, OH, USA

^g)^ Department of Environmental and Public Health Sciences, University of Cincinnati, Cincinnati, OH, USA

^i)^ Department of Electrical Engineering and Computer Science, University of Cincinnati, Cincinnati, OH, USA

^#^equal contribution

*Corresponding author: Leyla Esfandiari

**Email:** esfandla@ucmail.uc.edu

| Characterization technique | Young’s Modulus E | |
| --- | --- | --- |
|  | Soft (3 mg/mL Matrigel) | Stiff (12 mg/mL Matrigel) |
| Shear Rheology | 0.02893 $\pm$ 0.0018 kPa | 0.2485 $\pm$ 0.0390 kPa |
| Atomic Force Microscopy | 0.3183 $\pm$ 0.1547 | 0.8898 $\pm$ 0.1109 kPa |

**Table. S1.** **Mechanical Characterization of Soft and Stiff Hydrogels.**

Bulk (Rheology) and macroscopic (AFM)-level stiffness characterization of soft and stiff hydrogels. Data represented as mean $\pm$ SEM for n = 3 samples per group.

**Fig. S1.** **Imaging flow cytometry controls.**

Imaging flow cytometry controls showing gating strategy using unstained and negative controls including isotype IgG1 and IgG2b along with PBS + antibody samples for all three markers.

**Fig. S2.** **Super-resolution microscopy – biotinylated CD63 capture.**

Representative super-resolution microscopy images of single EVs captured using biotinylated CD63 and stained for Piezo1 or CD44 (scale bar = 50 nm).

**Fig. S3.** **Substrate stiffness does not drive differential Piezo1 in 2D culture-derived EVs.**

**a** Workflow indicating the experimental timeline and procedure for cell culture and small EV isolation. **b** Representative NTA graphs of purified EVs **c** Immunoblotting of Piezo1 and EV markers (TSG101, HSP70, CD63) in whole cell lysates and purified small EVs from Cal27 cells cultured on 0.5 kPa and 8 kPa substrates and Cal27 Piezo1-KO cells cultured on 0.5 kPa. Calnexin was used as a negative control. Molecular weights (in kDa) are shown to the right. Unprocessed blots are available as supplementary dataset S2. **d** Quantitative analysis of relative cellular Piezo1 levels for 0.5 kPa and 8 kPa substrates. Protein levels for 0.5 kPa were normalized as 1. Values are presented as mean ± SEM. n = 3 per group. Unpaired t-test. **e** Quantitative analysis of relative EV Piezo1 levels for 0.5 kPa and 8 kPa substrates. Protein levels for 0.5 kPa were normalized as 1. Values are presented as mean ± SEM. n = 3 per group. Unpaired t-test.

**Fig. S4.** **qRT-PCR of key differentially expressed miRNAs.**

qRT-PCR data for key differentially expressed miRNAs identified from miRNA-sequencing analysis. **a.** miR-146a-5p and **b.** miR-125a-5p were downregulated with stiffness, and **c.** miR-122-5p and **d.** miR-1246 were upregulated with stiffness. Values are presented as mean ± SEM. n = 3 per group. Unpaired t-test.

**Fig. S5. Recipient spheroid size in response to stiffness-primed EV treatment**

Temporal evolution of spheroid area over ten days in culture for all three conditions. Each solid mark represents the average of at least n = 100 spheroids with SEM. Values are presented as mean ± SEM. One-way ANOVA. *p < 0.05, **p < 0.01, ***p < 0.001, ****p < 0.0001. Asterisk color chart for comparisons: Pink (Untreated vs EV_soft_), Violet (Untreated vs EV_stiff_), and Green (EV_soft_ vs EV_stiff_).

a. **HSP70**

b. **TSG101**

c. **CD63**

d. **CD44**

e. **Piezo1**

f. **Calnexin**

Supplementary Dataset S1. **Immunoblotting raw data for spheroid lysates and spheroid-derived EVs.**

Raw western blot data for EV-specific markers **a** HSP70 **b** TSG101 **c** CD63), new markers of interest **d** CD44 and **e** Piezo1, and negative EV control **f** Calnexin for day 14 spheroid lysate and purified EVs samples for both soft and stiff matrix groups.

a. **HSP70**

b. **TSG101**

c. **CD63**

d. **Piezo1**

e. **Calnexin**

Supplementary Dataset S2. **Immunoblotting raw data for 2D cell lysates and cell culture-derived EVs.**

Raw western blot data for EV-specific markers **a** HSP70 **b** TSG101 **c** CD63, new marker of interest **d** Piezo1 and **e** negative EV control (Calnexin) for cell lysate and purified EVs samples for both 0.5 kPa and 8 kPa groups.

**2	3	4	5	7	8	9	11	12	13	16	17**

**Clone 2**: Might be homozygous KO or a larger deletion in the second allele.

CCACACCTGTCCTCTTGGTCTGACCGCGGCTGCTCCCTGCTCTCCGCAG**GCTGGACCTGAAGGACATCCCCAACGCCATCCGGC**

(CGGATGGAACTGCAGGCTGGGCCCCGGGGTGGGATTTGGAACTTCCTCCTCTCTTTAACACCAACTTACAACCAAAACGTCTACATCTGTTTTAAAAAGATTAAAATAACCCTCAAAATGGCTGAGACNACAGCTCTCGAATGACCTCCCTGACACACGGGTGACGCAGCTGCCCTCAC**CAGCTCCCGTGGATGTGGGCTCTGCCGGGTGTTCCTTGCAAGGCGCCCGCAGATGCCGAGGCAGACAGAGGAGACCACCAAGATGCCCAGGTCAGGGGCCACCA) inverted**

TTCAGACCTGTTTCTACTCGGGCTCTGCCTCTAGTCAGAAACCTCCACGCCCCGACATGGCATcTGTGCCCTTAGGAACTCTTCACAGG

**Clone 14:** Might be homozygous KO or a larger deletion in the second allele.

CCACACCTGTCCTCTTGGTCTGACCGCGGCTGCTCCCTGCTCTCCGCAG**GCTGGACCTGAAGGACATCCCCAA**

**~~CGCCATCCGGCTGGTGGCCCCTGACCTGGGCATCTTGGTGGTCTCCTCTGTCTGCCTCGGCATCTGCGGGCGCCTTGCAAGGAACACCCGGCAGAGCCCACATCCACGGGAGCTG~~**~~GTGAGGGCAGCTGCGTCACCCGTGTGTCAGGGAGGTCATTCGAGAGCTGTGGTCTCAGCCATTTTGAGGGTTATTTTAATCTTTTTAAAACAGATGTAGACGTTTTGGTTGTAAGTTGGTGTTAAAGAGAGGAGGAAGTTCCAAATCCCACCCCGGGGCCCAGCCTGCAGTTCCATCCGTTCAG~~

ACCTGTTTCTACTCGGGCTCTGCCTCTAGTCAGAAACCTCCACGCCCCGACATGGCATcTGTGCCCTTAGGAACTCTTCACAGG

**Clone 16:** Homozygous KO

Allele 1: KO	CCACACCTGTCCTCTTGGTCTGACCGCGGCTGCTCCCTGCTCTCCGCAG**GCTGGACCTGAAGGACATCCCCAACGCCATCCGGC**

(CGGATGGAACTGCAGGCTGGGCCCCGGGGTGGGATTTGGAACTTCCTCCTCTCTTTAACACCAACTTACAACCAAAACGTCTACATCTGTTTTAAAAAGATTAAAATAACCCTCAAAATGGCTGAGACNACAGCTCTCGAATGACCTCCCTGACACACGGGTGACGCAGCTGCCCTCAC**CAGCTCCCGTGGATGTGGGCTCTGCCGGGTGTTCCTTGCAAGGCGCCCGCAGATGCCGAGGCAGACAGAGGAGACCACCAAGATGCCCAGGTCAGGGGCCACCA) inverted**

TTCAGACCTGTTTCTACTCGGGCTCTGCCTCTAGTCAGAAACCTCCACGCCCCGACATGGCATcTGTGCCCTTAGGAACTCTTCACAGG

Allele 2: KO

CCACACCTGTCCTCTTGGTCTGACCGCGGCTGCTCCCTGCTCTCCGCAG**GCTGGACCTGAAGGACATCCCCAACGCCATCCGG**

**~~C~~**~~CGGATGG~~(aAACTGCAGGCTGGGCCCCGGGGTGGGATTTGGAACTTCCTCCTCTCTTTAACACCAACTTACAACCAAAACGTCTACATCTGTTTTAAAAAGATTAAAATAACCCTCAAAATGGCTGAGACNACAGCTCTCGAATGACCTCCCTGACACACGGGTGACGCAGCTGCCCTCAC**CAGCTCCCGTGGATGTGGGCTCTGCCGGGTGTTCCTTGCAAGGCGCCCGCAGATGCCGAGGCAGACAGAGGAGACCACCAAGATGCCCAGGTCAGGGGCCACCA) inverted**

TTCAGACCTGTTTCTACTCGGGCTCTGCCTCTAGTCAGAAACCTCCACGCCCCGACATGGCATcTGTGCCCTTAGGAACTCTTCACAGG

Supplementary Dataset S3. **Genotyping validation of Piezo1-KO cell cloning.**

Genotyping of Piezo1-KO Cal27 cells. Clones 2, 4, 5, 7, 14, 15, 16, and 17 were picked for Sanger sequencing. Clones 2, 14, and 16 were confirmed to have the presence of a knockout allele. Clone 2 cells were utilized for all KO group experiments.

Supplementary Dataset S4. **Immunoblotting raw data for recipient spheroids treated with stiffness-primed EVs. R**aw western blot data for proliferation markers Ki67 and SOX2, and the apoptosis-related marker Caspase3, with β-actin as a loading control, in recipient spheroids across all treatment conditions.
